# Biomarker Analysis in Upper Respiratory Tract Infections: Associations with Demographics and Clinical Outcomes

**DOI:** 10.3390/pediatric17010001

**Published:** 2024-12-26

**Authors:** Felicia Manole, Alexia Manole, Andrei Nicolae Csep, Lavinia Davidescu, Călin Tudor Hozan, Eduard Szilagy, Florica Voiță-Mekeres, Andrada Florina Schwarz-Madar, Ariana Szilagyi

**Affiliations:** 1 Faculty of Medicine and Pharmacy, University of Oradea, 410087 Oradea, Romania; fmanole@uoradea.ro (F.M.); alexia.manole@student.uoradea.ro (A.M.); lavinia.davidescu@didactic.uoradea.ro (L.D.); chozan@uoradea.ro (C.T.H.); mekeres_florina@yahoo.com (F.V.-M.); schwarz_andrada@yahoo.com (A.F.S.-M.); ariana.szilagyi@didactic.uoradea.ro (A.S.); 2 Faculty of Medicine, “George Emil Palade” University of Medicine, Pharmacy, Science and Technology of Targu Mures, 540142 Targu Mures, Romania; szilagyi.eduard.19@stud.umfst.ro

**Keywords:** upper respiratory tract infections, biomarkers, immunoglobulin A, immunoglobulin E, neutrophils, C-reactive protein

## Abstract

Background/Objectives: Upper respiratory tract infections (URTIs) are a significant global health burden, and understanding the immune response is crucial for developing effective diagnostic tools and treatment strategies. Methods: This study investigated the levels of specific biomarkers in 188 patients with URTIs and their association with demographic factors, comorbidities, and clinical outcomes. Immunoglobulin A (IgA), immunoglobulin E (IgE), neutrophils, serum iron, C-reactive protein (CRP), and erythrocyte sedimentation rate (ESR) were measured. Results: The median age of the patients was 5 years, with 46% being female and 63% from urban areas. Adenoiditis (37%), otitis (25%), and rhinitis (20%) were the most common diagnoses. While most biomarkers did not vary significantly by gender, neutrophil levels were significantly higher in females (*p* = 0.020). IgE levels were significantly elevated in rural patients compared to urban counterparts (*p* = 0.034). Conclusions: ESR was significantly associated with rhinitis diagnosis, and IgE and ESR were predictive of otitis in multivariate models. However, many biomarkers did not significantly correlate with other diagnoses, contradicting previous research focusing on individual biomarkers. This study highlights the complexity of immune responses in URTIs and the need for more effective diagnostic tools. The findings can inform the development of tailored treatment strategies based on gender, area of origin, and infection type.

## 1. Introduction

Upper respiratory tract infections (URTIs) are indeed a widespread health concern, affecting a significant portion of the global population. They are one of the leading causes of hospital visits worldwide and account for a substantial number of outpatient consultations [1,2].

Particularly common in children, URTIs account for a substantial percentage of pediatric cases, contributing to increased healthcare utilization and parental absenteeism from work [3,4].

While often considered mild and self-limiting, URTIs can significantly disrupt patients’ daily activities and quality of life. A multi-country study revealed that URTIs exert a considerable deleterious effect on sufferers, prompting many to seek medical attention and medication for relief [5]. This contrasts the common perception of URTIs as merely a nuisance and underscores the importance of effective management strategies to improve patient outcomes and reduce healthcare costs.

Despite their high prevalence, the management of URTIs poses significant challenges, particularly regarding antibiotic-prescribing practices. Although most URTIs are viral in origin and resolve naturally, there remains a tendency for the overprescription of antibiotics, contributing to the growing problem of antimicrobial resistance [6,7,8].

This issue highlights the need for improved patient education, adherence to evidence-based guidelines, and targeted interventions to promote the judicious use of antibiotics in URTI management [3,7].

Research into preventive measures and treatments for URTIs has yielded mixed results. For instance, vitamin D supplementation, hypothesized to reduce URTI-related health service utilization, showed no significant association in a cohort study of children [9]. Conversely, probiotics have demonstrated promise in reducing the number and duration of URTI episodes and secondary complications in URTI-prone children [9]. Iron supplementation has also been found to improve the iron status and reduce morbidity from URTIs in pediatric populations [10]. These findings suggest that while some interventions may be beneficial for specific groups, more research is needed to identify consistently effective preventive measures and treatments.

Previous studies have investigated the epidemiology, pathogenesis, and treatment of URTIs, yet a comprehensive understanding of the underlying mechanisms and reliable biomarkers remains lacking. URTIs can be caused by various pathogens, including viruses and bacteria, with the oropharynx and nasopharynx typically colonized by polymicrobial flora [11,12]. Interestingly, most URTIs resolve naturally, even when bacteria are the causative agents, challenging the widespread use of antibiotics for these conditions [13]. Additionally, the introduction of pneumococcal conjugate vaccines has altered the relative frequency of major bacterial pathogens causing acute otitis media and acute bacterial rhinosinusitis, further complicating the clinical landscape [14].

Biomarkers play a crucial role in the early diagnosis and management of infections, especially for patients with comorbidities or at a high risk of complications. C-reactive protein (CRP) has shown potential as a marker for predicting the severity of URTIs, with studies finding strong correlations between serum CRP levels and clinical indicators, such as the body temperature and white blood cell counts [15]. However, other biomarkers, like procalcitonin (PCT), while promising for severe bacterial infections, have not been proven suitable for less severe or localized infections, like URTIs [15,16,17]. This limitation underscores the need for more specific biomarkers tailored to URTIs to enhance the diagnostic accuracy and guide appropriate treatment strategies.

The immune response to URTIs can be further understood by examining specific biomarkers, such as immunoglobulins and inflammatory cells. Immunoglobulin A (IgA) and immunoglobulin E (IgE) are particularly relevant; IgA plays a key role in mucosal immunity, while IgE is associated with allergic reactions and certain infections. Neutrophils, as essential components of the innate immune system, serve as indicators of infection and inflammation and may provide insights into the severity and progression of URTIs.

Given the significant morbidity associated with URTIs—including conditions like adenoiditis, otitis media, rhinitis, and sinusitis—and the complex challenges they present in diagnosis, management, and prevention, understanding the immune response in these infections is essential. Investigating specific biomarkers, such as IgA, IgE, and neutrophils, may offer valuable information for early diagnosis and the development of targeted treatment strategies.

The aim of this study is to investigate the levels of specific biomarkers—namely IgA, IgE, and neutrophils—in patients with URTIs and to determine their associations with demographic factors, comorbidities, and clinical outcomes.

## 2. Materials and Methods

### 2.1. Data Collection

A total of 195 patients diagnosed with upper respiratory tract infections (URTIs)—including adenoiditis, otitis media, rhinitis, and sinusitis—were enrolled in the study. Demographic details, such as age, gender, and area of origin, were collected. Levels of specific biomarkers were measured, including immunoglobulin A (IgA), immunoglobulin E (IgE), neutrophil counts, serum iron levels, erythrocyte sedimentation rate (ESR), and C-reactive protein (CRP). These biomarkers were selected for their ability to provide complementary insights into the immune and inflammatory responses associated with URTIs. Together, they capture a spectrum of immune dynamics, from specific antibody responses (IgA, IgE) and acute inflammation (CRP, ESR) to cellular defense mechanisms (neutrophils) and systemic metabolic adaptations (serum iron). This multifaceted approach enables a comprehensive understanding of the immune mechanisms underlying URTIs and helps to identify potential predictors of clinical outcomes, tailored to pediatric patients’ unique immunological profiles.

### 2.2. Inclusion and Exclusion Criteria

Patients eligible for inclusion were children under 18 years of age who presented with clinical symptoms consistent with an acute URTI, confirmed based on diagnostic criteria specific to otorhinolaryngology (ORL) practice. Only those with a recent onset of symptoms—within the past 14 days—were considered, focusing on acute presentations and minimizing variability due to chronic conditions. Additionally, patients had not received antibiotic or systemic anti-inflammatory treatments for their current infection, ensuring that natural immune response markers were not influenced by prior medication. All participants provided informed consent, confirming their willingness and ability to partake in the study.

Patients were excluded if they had chronic or recurrent upper respiratory infections, as these cases may exhibit different immune profiles compared to acute cases. Those with malignant hematologic conditions or complex malformation syndromes or those who had undergone treatment for malignancies in the past two months were also excluded. Individuals undergoing immunosuppressive therapy or with known immunodeficiency disorders were not included due to potential alterations in biomarker levels. Patients with underlying chronic respiratory diseases, such as chronic obstructive pulmonary disease (COPD) or asthma, were omitted to prevent confounding immune responses. Cases where upper respiratory symptoms were attributed to allergies rather than infections or where diagnoses were inconclusive were not included. Furthermore, individuals with concurrent systemic infections or severe comorbidities that could independently influence inflammatory or immune markers were excluded from the study.

### 2.3. Ethical Approval

The study was approved by the Ethics Board of Bihor County Emergency Hospital on 14 March 2024, with approval number 8271/14.03.2024.

### 2.4. Statistical Analysis

Data processing, centralization, and analysis were conducted using RStudio (compatible with R version 4.4.1). Microsoft Excel was utilized for initial data storage and organization.

The original sample size was N = 195; after data processing and outlier elimination, the final sample size was N = 188.

Descriptive statistics for nominal data were reported as frequencies and percentages. For continuous variables following a normal distribution, data were presented as the mean ± standard deviation (SD); for non-normally distributed data, results were reported as the median with the interquartile range (IQR). The normality of continuous data was assessed using the Shapiro–Wilk test.

To compare differences between groups, statistical tests appropriate for the data distribution were employed. For normally distributed data, the independent samples *t*-test was used to compare means between two groups, while the Kruskal–Wallis H test was applied for non-parametric comparisons among multiple groups.

To assess the relationships between specific biomarkers (independent variables) and diagnosis categories (dependent variable), appropriate statistical analyses were conducted. Given that the diagnosis is a categorical variable with multiple categories (e.g., adenoiditis, otitis media, rhinitis, sinusitis), an analysis of variance (ANOVA) or the Kruskal–Wallis test was used to identify significant differences in biomarker levels across different diagnoses.

Following univariate analyses, multivariate regression models were constructed to identify independent predictors of the diagnosis. Multinomial logistic regression was employed due to the categorical nature of the dependent variable with more than two categories. The models were optimized using forward and backward selection methods to identify the most significant predictors among the biomarkers.

In this study, the independent variables included immunoglobulin A (IgA), immunoglobulin E (IgE), neutrophil counts, serum iron levels, erythrocyte sedimentation rate (ESR), and C-reactive protein (CRP). The dependent variable was the diagnosis category.

A significance level of *p* < 0.05 was set as the threshold for statistical significance (alpha = 0.05) throughout the study. Various plots and graphical representations were generated to visually illustrate key findings and relationships, as well as to validate statistical assumptions.

## 3. Results

Our study had 188 patients with the median age of 5 years, of whom 86 (46%) were female. About 63% of the patients come from the urban area (Table 1).

More than a third of the patients were admitted in the study with a diagnosis of adenoiditis (37%). The second most frequent diagnosis was otitis (25%), followed by rhinitis (20% of the diagnosed cases). Out of the total sample population, 26 were diagnosed with sinusitis (14%) and 8 suffered from tonsillitis (4%).

Levels of specific biomarkers were closely monitored, and it was established that immunoglobulin A had a median of 98 mg/dL, immunoglobulin E levels had a median of 20 kU/L, and neutrophils, at 3.30 ×109/L, were all normal levels for the age category studied. Serum iron levels concentrated around the median of 79 mcg/dL, the C-reactive protein (CPR) was around the value of 1.00 mg/L, and the erythrocyte sedimentation rate had a median of 8 mm/h (Table 2).

**Figure 1:**
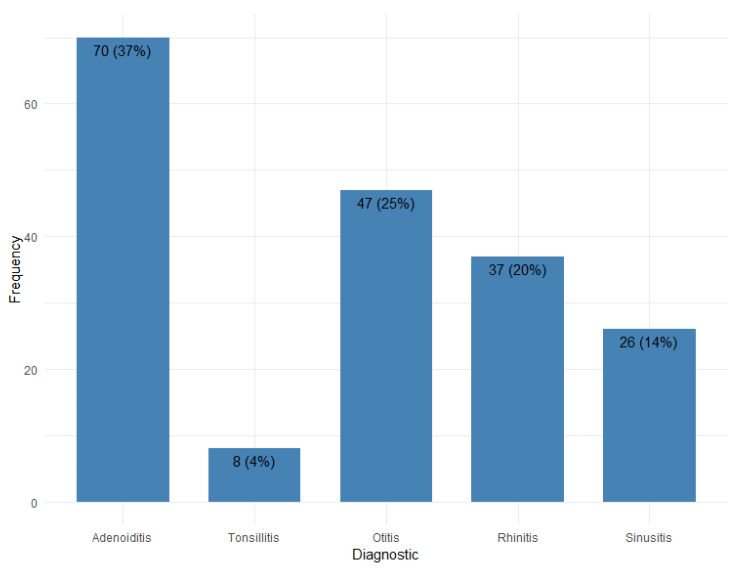
Frequency of diagnosis for the sample size.

The demographic and clinical characteristics of female and male participants were analyzed. The median age for both genders of participants was 5 years. Although there was a higher proportion of male participants from both rural and urban areas, the difference was not statistically significant (*p*-value = 0.654). Males exhibited a higher median IgA level, but this difference was not statistically significant (*p*-value = 0.882). Immunoglobulin E, serum iron, CPR, and ESR levels demonstrated no gender-related differences (IgE *p*-value = 0.940, serum iron *p*-value = 0.255, CPR *p*-value = 0.334, ESR *p*-value = 0.570). The sole clinical variable that exhibited gender-related differences was the neutrophil level. Neutrophil levels were significantly higher in the female population compared to males (*p*-value = 0.020). Despite variations in the frequencies of different types of upper respiratory tract infections between males and females, there was insufficient statistical evidence to conclude significance (*p*-values > 0.05) (Table 3, Figure 1).

**Figure 2:**
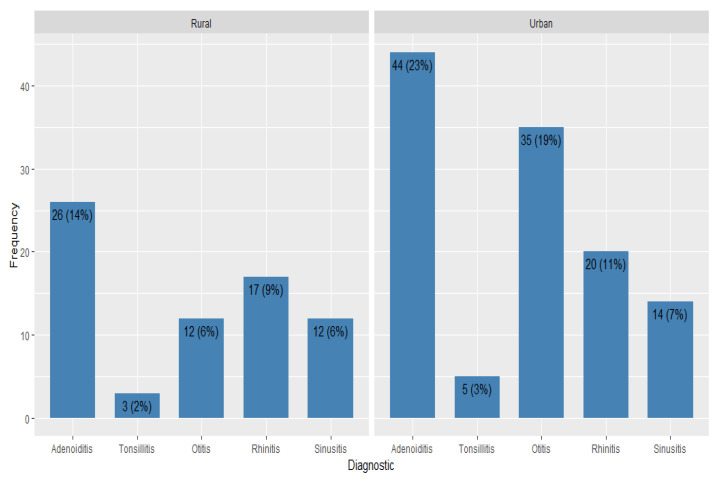
Distribution of diagnoses by patient origin.

The difference in demographic and clinical characteristics between participants originating in the rural and urban area was then studied. Patients from the rural area had a higher median IgA level, but not significantly (*p*-value = 0.319). Neutrophils, serum iron, CPR, and ESR levels showed no area-related differences (Neu *p*-value = 0.198, serum iron *p*-value = 0.411, CPR *p*-value = 0.424, ESR *p*-value = 0.460). The only clinical variable that showed area-related differences was the immunoglobulin E level. IgE levels were higher in male population than females (*p*-value = 0.034). Even though the frequencies of the different types of upper respiratory tract infections varied between males and females, there was no statistical evidence to conclude significance (*p*-values > 0.05). (Table 4, Figure 2).

**Figure 3:**
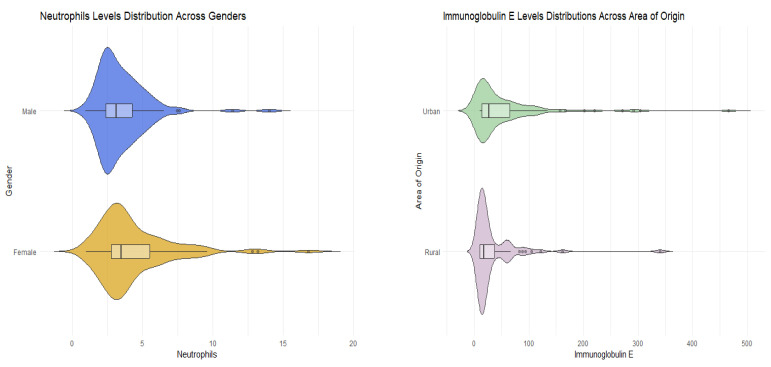

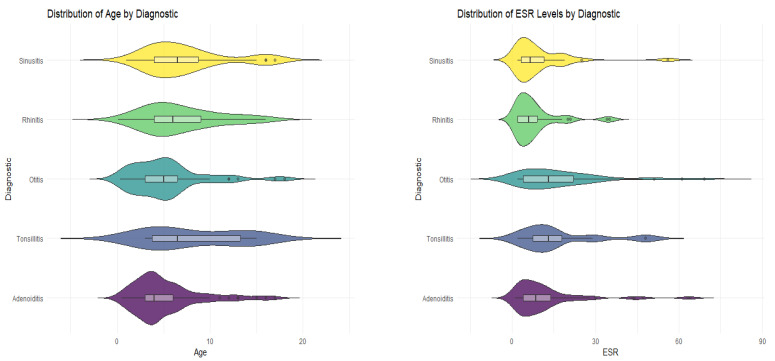
Graphical representations (violin plots) of key relationships between the studied characteristics.

The difference in demographic and clinical characteristics between participants considering the given diagnosis was then studied. Statistical tests showed that there was no significant difference in levels of IgA, neutrophils, and serum iron in the different groups of diagnosis (*p*-values > 0.05) and that there was also no difference in proportions for gender and the area of origin. Significant differences between the medians of the diagnostic groups were found in several key characteristics, such as age, IgE, CPR, and ESR. Patients diagnosed with tonsillitis and sinusitis were generally older with a median age of 6.5 years. Patients who received the diagnosis of tonsillitis had a considerably higher level of immunoglobulin E, a median of 50 IU/mL, the highest levels of C-reactive protein (median of 5.50 mg/L), and an erythrocyte sedimentation rate of 13 mm/h (Table 5, Figure 3).

Regression analysis between adenoiditis and the specific biomarkers (Table 6) showed that no explanatory variable was effective alone in predicting the probability of the result, as all the *p*-values for the univariate regression models were higher than 0.05. There was also no success in predicting the probability using a multivariate regression model built with the backward selection method, with the final model containing only the intercept and having a null R^2^ coefficient, which highlights an inefficient model. 

The univariate logistic regression model showed that CRP has a statistically significant effect in predicting otitis (*p*-value = 0.011) with every unit increase in the CRP levels resulting in a 3% increase in the odds of being diagnosed with otitis. A statistically significant relationship was found between the diagnosis of otitis and immunoglobulin E and CRP levels (Table 7).

The analysis demonstrated that CRP was the only variable found to be statistically significant in both the univariate and multivariate regression models. In the univariate regression, CRP showed a significant association with rhinitis, with an odds ratio (OR) of 0.95 (95% confidence interval [CI]: 0.91–0.99, *p* = 0.035) which indicated that for every unit increase in CRP (measured in mg/dL), the odds of having rhinitis decrease by approximately 5%, assuming that all other factors remain constant.

After applying the backward selection method in the multivariate regression model, CRP was the only explanatory variable retained. The association remained statistically significant, with the same odds ratio (0.95) and confidence interval (95% CI: 0.91–0.99, *p* = 0.035), highlighting CRP as a key predictor of rhinitis in our model.

Other variables, including IgA, neutrophils, IgE, serum iron, and ESR, were not statistically significant in either the univariate or multivariate regression models (Table 8).

The univariate regression analysis for the diagnosis of sinusitis revealed that none of the explanatory variables were statistically significant predictors.

Specifically, IgA had an OR of 1.00 (95% CI: 0.99–1.01, *p* > 0.9), suggesting no effect on the odds of sinusitis. Neutrophils showed a slightly elevated OR of 1.05 (95% CI: 0.87–1.22, *p* = 0.6), but this was not statistically significant. IgE also had no significant association, with an OR of 1.00 (95% CI: 0.99–1.01, *p* = 0.8). Similarly, serum iron and ESR displayed ORs of 1.00 (95% CI: 0.99–1.01, *p* = 0.7) and 0.98 (95% CI: 0.93–1.01, *p* = 0.3), respectively, with no evidence of statistical significance.

CRP showed an OR of 0.96 (95% CI: 0.83–1.03, *p* = 0.5), suggesting a potential negative association, but this was not statistically significant either (Table 9 and Table 10).

The univariate regression analysis for the diagnosis of tonsillitis indicates that none of the explanatory variables are statistically significant predictors of the condition.

For IgA, the OR was 1.00 (95% CI: 0.99–1.01, *p* = 0.5), indicating no detectable effect on the likelihood of tonsillitis. Neutrophils had an OR of 0.95 (95% CI: 0.62–1.24, *p* = 0.8), showing no significant association. IgE presented with an OR of 1.00 (95% CI: 0.99–1.01, *p* = 0.6), similarly indicating no effect.

Serum iron displayed a slightly elevated OR of 1.01 (95% CI: 0.98–1.02, *p* = 0.6), which was not statistically significant. ESR had an OR of 1.02 (95% CI: 0.97–1.06, *p* = 0.4), and CRP showed an OR of 1.02 (95% CI: 0.92–1.07, *p* = 0.6), but neither variable demonstrated a significant association with the diagnosis of tonsillitis.

## 4. Discussion

The present study aimed to investigate the levels of specific biomarkers in patients with upper respiratory tract infections (URTIs) and to examine their associations with demographic factors, comorbidities, and clinical outcomes. Our findings offer new insights into how gender and the geographical origin may influence immune responses in URTIs.

One of the key observations was that neutrophil levels were significantly higher in female patients. This aligns with earlier studies by Gupta et al. and Sumera et al., who suggested that gender-related differences in immune responses may be attributed to hormonal influences or genetic predispositions leading to higher neutrophil counts in women [7,18]. Understanding this gender-specific variation is crucial for clinicians when interpreting laboratory results and could inform more personalized approaches to managing URTIs in female patients.

Another significant finding was that patients from rural areas had higher IgE levels, aligning with literature suggesting that increased allergen exposure in these environments may influence IgE-related immune responses [13,19,20,21].

This geographical difference underscores the importance of considering environmental exposures when evaluating IgE levels and could have implications for the diagnosis and management of allergic conditions associated with URTIs.

Contrary to some previous research, our study found that many biomarkers did not significantly correlate with specific diagnoses. For instance, while elevated IgE levels have been associated with allergic rhinitis in other studies [22], we did not observe a significant relationship in our cohort. This discrepancy may reflect population-specific variations in immune responses or unaccounted environmental or genetic factors influencing IgE’s role in URTIs.

Similarly, although neutrophil levels have been implicated in the diagnosis of sinusitis [23], our results did not demonstrate a significant correlation, suggesting that neutrophil elevation may not be universally applicable as a diagnostic marker for sinusitis across diverse populations.

The erythrocyte sedimentation rate (ESR) emerged as a significant predictor of rhinitis and, along with IgE levels, was a significant predictor of otitis in multivariate models. Elevated ESR levels are typically associated with bacterial and fungal infections, providing valuable insight for an early diagnosis [24,25,26]. This finding suggests that ESR, a readily available and cost-effective test, could play a crucial role in distinguishing between different types of URTIs and guiding appropriate treatment strategies. Moreover, ESR has been shown to be an important factor in determining the treatment response, emphasizing its clinical utility [27].

Our study differs from previous research by simultaneously analyzing multiple biomarkers and exploring their associations with demographic factors, such as gender and the area of origin. This comprehensive approach highlights the complexity of immune responses in URTIs and emphasizes the need for more effective diagnostic tools that consider multiple factors rather than relying on individual biomarkers alone.

The findings of this study have important clinical implications. Recognizing that neutrophil levels are higher in females and that IgE levels are elevated in rural populations can aid clinicians in interpreting laboratory results more accurately and tailoring treatment plans accordingly. For example, elevated neutrophil counts in female patients may be considered a normal variation rather than an immediate cause for concern, whereas higher IgE levels in rural patients might prompt further investigation into environmental allergen exposure.

The significant predictive role of ESR in diagnosing rhinitis and otitis suggests that incorporating ESR measurements into routine diagnostic protocols could enhance early detection and improve patient outcomes. Since ESR is a simple and inexpensive test, its use in primary care settings could be particularly beneficial, especially in resource-limited environments.

While previous research has often focused on individual biomarkers, our study underscores the importance of a multifaceted approach to understanding immune responses in URTIs. The lack of significant correlations between certain biomarkers and specific diagnoses in our study suggests that the pathophysiology of URTIs may be more complex than previously thought. This complexity necessitates the development of more comprehensive diagnostic tools that can account for the interplay of multiple biomarkers and patient characteristics.

Our findings challenge some established notions, such as the universal applicability of neutrophil counts in diagnosing sinusitis or the direct association between IgE levels and allergic rhinitis. These contradictions highlight the variability of immune responses across different populations and emphasize the need for personalized medicine approaches in the management of URTIs.

### 4.1. Limitations

Several limitations should be considered when interpreting the results of this study. Firstly, the sample size was limited and drawn from a specific geographic region, which may affect the generalizability of the findings. The population consisted of young individuals, limiting the applicability of the results to older demographics. Additionally, the cross-sectional design of the study precludes the ability to establish causal relationships between biomarker levels and clinical outcomes.

Another limitation is the potential influence of unmeasured confounding factors, such as the socioeconomic status, nutritional status, or other environmental exposures that were not accounted for in the analysis. These factors could affect immune responses and biomarker levels, potentially impacting the study’s conclusions.

### 4.2. Future Directions

Future research should aim to validate these findings in larger, more diverse populations, including different age groups and geographical locations. Longitudinal studies could provide insights into how biomarker levels change over the course of infection and recovery, helping to establish temporal relationships and potential causality.

Investigating additional biomarkers, such as cytokines or vitamin D levels, could further elucidate the immune mechanisms involved in URTIs. For example, studies in other medical fields suggest that optimal vitamin D levels support tissue integrity and may influence inflammatory responses [28]. Exploring such connections could lead to novel diagnostic and therapeutic strategies.

Moreover, the high healthcare costs associated with aging populations—for example, the average U.S. elderly care cost was $48,101 in 2015—underscore the need for cost-effective strategies [29]. Implementing preventive approaches in URTI management, such as promoting home care, could reduce healthcare utilization and improve outcomes for high-risk groups like the elderly.

Additionally, methodological challenges, like small sample sizes and inadequate statistical reporting, similar to those found in cognitive-behavioral therapy research for childhood anxiety, highlight the importance of standardized research protocols [30]. Addressing these limitations may enhance the validity and applicability of findings in URTIs and other health conditions.

Moreover, integrating assessments of environmental exposures and genetic factors could enhance our understanding of individual variations in immune responses. This holistic approach may pave the way for personalized interventions that improve patient outcomes and reduce the burden of URTIs on healthcare systems.

## 5. Conclusions

This study contributes to the growing body of evidence on the complex immune responses associated with upper respiratory tract infections. By highlighting significant associations between specific biomarkers and demographic factors, it underscores the importance of considering gender and geographical origin in the diagnosis and management of URTIs.

The findings suggest that relying solely on individual biomarkers may not be sufficient for an accurate diagnosis, emphasizing the need for comprehensive diagnostic tools that incorporate multiple biomarkers and patient characteristics. Such tools could lead to more effective and tailored treatment strategies, ultimately improving patient care.

While this study offers valuable insights, some associations—such as between IgE levels and the area of residence—are relatively weak and should be interpreted with caution. Nevertheless, the study lays the groundwork for developing more personalized and effective approaches to diagnosing and managing upper respiratory tract infections. Further research is needed to validate these findings, explore additional biomarkers, and refine diagnostic criteria for improved clinical outcomes.

## Figures and Tables

**Table 1:** Demographic measurement of patients.

Variable	N = 188 (100%) ^1^
Age	5.0 (3.0, 7.0)
Gender	
Female	86 (46%)
Male	102 (54%)
Area of origin	
Rural	70 (37%)
Urban	118 (63%)

^1^ Results are presented as the median (percentile 25th, percentile 75th) and n (%).

**Table 2:** Baseline characteristics of patients.

Variable	N = 188 (100%) ^1^
IgA (mg/dL)	98 (66, 148)
Neutrophils (×10^9^/L)	3.30 (2.50, 4.64)
IgE (IU/mL)	20 (12, 58)
Serum Iron (µg/dL)	79 (56, 100)
CPR (mg/dL)	1.00 (1.00, 2.85)
ESR (mm/h)	8 (4, 16)

^1^ Results are presented as the median (percentile 25th, percentile 75th) and n (%). IgA: immunoglobulin A, IgE: immunoglobulin E, Neu: neutrophils, CPR: C-reactive protein, ESR: erythrocyte sedimentation rate.

**Table 3:** Comparative analysis of demographic and clinical variables by gender in patient cohort.

Variable	Female, N = 86 (46%) ^1^	Male, N = 102 (54%) ^1^	*p*-Value ^2^
Age (years)	5.0 (3.0, 8.0)	5.0 (3.0, 7.0)	0.080
Area of origin			
Rural	34 (40%)	36 (35%)	0.654
Urban	52 (60%)	66 (65%)	0.654
IgA (mg/dL)	94 (65, 150)	98 (67, 147)	0.882
Neutrophils (×10^9^/L)	3.47 (2.78, 5.60)	3.12 (2.38, 4.27)	0.020
IgE (IU/mL)	22 (13, 59)	19 (10, 58)	0.940
Serum Iron (µg/dL)	77 (53, 97)	80 (59, 104)	0.255
CPR (mg/dL)	1.05 (1.00, 4.00)	1.00 (1.00, 1.80)	0.344
ESR (mm/h)	8 (4, 14)	9 (4, 17)	0.570
Diagnosis			
Adenoiditis	33 (38%)	37 (36%)	0.885
Tonsilitis	6 (7.0%)	2 (2.0%)	0.182
Sinusitis	10 (12%)	16 (16%)	0.555
Rhinitis	15 (17%)	22 (22%)	0.600
Otitis	22 (26%)	25 (25%)	>0.999

^1^ Results are presented as the median (percentile 25th, percentile 75th) and n (%). ^2^ Welch two sample *t*-test; standardized mean difference; 2-sample test for equality of proportions with continuity correction. IgA: immunoglobulin A, IgE: immunoglobulin E, Neu: neutrophils, CPR: C-reactive protein, ESR: erythrocyte sedimentation rate.

**Table 4:** Comparative analysis of demographic and clinical variables by area of origin in patient cohort.

Variable	Rural, N = 70 (37%) ^1^	Urban, N = 118 (63%) ^1^	*p*-Value ^2^
Age	5.0 (3.0, 7.0)	5.0 (3.0, 8.0)	0.579
Gender			
Female	34 (49%)	52 (44%)	0.654
Male	36 (51%)	66 (56%)	0.654
IgA (mg/dL)	114 (68, 156)	91 (64, 144)	0.319
Neutrophils (×10^9^/L)	3.42 (2.56, 5.29)	3.23 (2.44, 4.27)	0.198
IgE (IU/mL)	17 (10, 37)	27 (14, 66)	0.034
Serum Iron (µg/dL)	80 (58, 99)	78 (55, 100)	0.411
CPR (mg/dL)	1.00 (1.00, 2.50)	1.00 (1.00, 3.00)	0.424
ESR (mm/h)	10 (6, 15)	7 (2, 16)	0.460
Diagnosis			
Adenoiditis	26 (37%)	44 (37%)	>0.999
Tonsillitis	3 (4.3%)	5 (4.2%)	>0.999
Sinusitis	12 (17%)	14 (12%)	0.427
Rhinitis	17 (24%)	20 (17%)	0.301
Otitis	12 (17%)	35 (30%)	0.082

^1^ Results are presented as the median (percentile 25th, percentile 75th) and n (%). ^2^ Welch two sample *t*-test; standardized mean difference; 2-sample test for equality of proportions with continuity correction. IgA: immunoglobulin A, IgE: immunoglobulin E, Neu: neutrophils, CPR: C-reactive protein, ESR: erythrocyte sedimentation rate.

**Table 5:** Comparative analysis of demographic and clinical variables by diagnostic in patient cohort.

Variable	Adenoiditis, N = 70 (37%) ^1^	Tonsillitis, N = 8 (4.3%) ^1^	Otitis, N = 47 (25%) ^1^	Rhinitis, N = 37 (20%) ^1^	Sinusitis, N = 26 (14%) ^1^	*p*-Value ^2^
Age (Years)	4.0 (3.0, 6.0)	6.5 (3.5, 13.5)	5.0 (3.0, 7.0)	6.0 (4.0, 9.0)	6.5 (4.0, 9.0)	0.032
Gender						
Female	33 (47%)	6 (75%)	22 (47%)	15 (41%)	10 (38%)	0.437
Male	37 (53%)	2 (25%)	25 (53%)	22 (59%)	16 (62%)	0.437
Area of origin						
Rural	26 (37%)	3 (38%)	12 (26%)	17 (46%)	12 (46%)	0.307
Urban	44 (63%)	5 (63%)	35 (74%)	20 (54%)	14 (54%)	0.307
IgA (mg/dL)	90 (58, 152)	108 (84, 148)	90 (64, 147)	102 (76, 142)	103 (78, 134)	0.892
Neutrophils (×10^9^/L)	3.30 (2.47, 4.59)	3.09 (2.54, 4.59)	3.38 (2.52, 5.18)	3.18 (2.17, 4.05)	3.71 (2.44, 5.29)	0.804
IgE (IU/mL)	25 (14, 71)	50 (17, 78)	15 (10, 31)	20 (10, 63)	24 (16, 58)	0.026
Serum Iron (µg/dL)	76 (50, 97)	86 (52, 117)	80 (53, 109)	78 (60, 99)	81 (59, 93)	0.760
CPR (mg/dL)	1.00 (1.00, 2.30)	5.50 (2.75, 7.20)	1.00 (1.00, 3.60)	1.00 (1.00, 1.50)	1.00 (1.00, 2.00)	0.028
ESR (mm/h)	9 (4, 14)	13 (7, 22)	13 (4, 22)	6 (2, 9)	7 (3, 12)	0.011

^1^ Results are presented as the median (percentile 25th, percentile 75th) and n (%). ^2^ Kruskal–Wallis rank sum test. IgA: immunoglobulin A, IgE: immunoglobulin E, Neu: neutrophils, CPR: C-reactive protein, ESR: erythrocyte sedimentation rate.

**Table 6:** Comparisons of univariate and multivariate models (built with the backward selection method) for adenoiditis.

Diagnostic: Adenoiditis	Univariate Regression			
Explanatory Variable	N	OR ^1^	95% CI ^1^	*p*-Value
IgA (mg/dL)	188	1.00	0.99, 1.00	0.6
Neutrophils (×10^9^/L)	188	1.02	0.89, 1.15	0.8
IgE (IU/mL)	188	1.00	1.00, 1.01	0.6
Serum Iron (µg/dL)	188	1.00	0.99, 1.00	0.3
CPR (mg/dL)	188	1.00	0.97, 1.02	0.8
ESR (mm/h)	188	0.99	0.94, 1.02	0.5

^1^ Odds Ratios (OR) and 95% Confidence Intervals (CI) to assess the association of diagnostic variables with adenoiditis and otitis.

**Table 7:** Comparisons of univariate and multivariate models (built with the backward selection method) for otitis.

Diagnostic: Otitis	Univariate Regression				Multivariate Regression		
Explanatory Variable	N	OR ^1^	95% CI ^1^	*p*-Value	OR ^1^	95% CI ^1^	*p*-Value
IgA (mg/dL)	188	1.00	1.00, 1.01	0.7			
Neutrophils (×10^9^/L)	188	1.06	0.92, 1.20	0.4			
IgE (IU/mL)	188	0.99	0.98, 1.00	0.057	0.99	0.98, 1.00	0.038
Serum Iron (µg/dL)	188	1.00	0.99, 1.01	0.6			
CPR (mg/dL)	188	1.03	1.01, 1.06	0.011	1.03	1.01, 1.06	0.006
ESR (mm/h)	188	1.02	0.98, 1.06	0.3			

^1^ OR = odds ratio, CI = confidence interval. IgA: immunoglobulin A, IgE: immunoglobulin E, Neu: neutrophils, CPR: C-reactive protein, ESR: erythrocyte sedimentation rate.

**Table 8:** Comparisons of univariate and multivariate models (built with the backward selection method) for rhinitis.

Diagnostic: Rhinitis	Univariate Regression				Multivariate Regression		
Explanatory Variable	N	OR ^1^	95% CI ^1^	*p*-Value	OR ^1^	95% CI ^1^	*p*-Value
IgA (mg/dL)	188	1.00	0.99, 1.01	0.9			
Neutrophils (×10^9^/L)	188	0.85	0.68, 1.02	0.12			
IgE (IU/mL)	188	1.00	1.00, 1.01	0.3			
Serum Iron (µg/dL)	188	1.00	0.99, 1.01	0.4			
CPR (mg/dL)	188	0.95	0.91, 0.99	0.035	0.95	0.91, 0.99	0.035
ESR (mm/h)	188	1.00	0.94, 1.04	>0.9			

^1^ OR = odds ratio, CI = confidence interval. IgA: immunoglobulin A, IgE: immunoglobulin E, Neu: neutrophils, CPR: C-reactive protein, ESR: erythrocyte sedimentation rate.

**Table 9:** Comparisons of univariate and multivariate models (built with the backward selection method) for sinusitis.

Diagnostic: Sinusitis	Univariate Regression			
Explanatory Variable	N	OR ^1^	95% CI ^1^	*p*-Value
IgA (mg/dL)	188	1.00	0.99, 1.01	>0.9
Neutrophils (×10^9^/L)	188	1.05	0.87, 1.22	0.6
IgE (IU/mL)	188	1.00	0.99, 1.01	0.8
Serum Iron (µg/dL)	188	1.00	0.99, 1.01	0.7
ESR (mm/h)	188	0.98	0.93, 1.01	0.3
CPR (mg/dL)	188	0.96	0.83, 1.03	0.5

^1^ OR = odds ratio, CI = confidence interval. IgA: immunoglobulin A, IgE: immunoglobulin E, Neu: neutrophils, CPR: C-reactive protein, ESR: erythrocyte sedimentation rate.

**Table 10:** Comparisons of univariate and multivariate models (built with the backward selection method) for tonsilitis.

Diagnostic: Tonsillitis	Univariate Regression			
Explanatory Variable	N	OR ^1^	95% CI ^1^	*p*-Value
IgA (mg/dL)	188	1.00	0.99, 1.01	0.5
Neutrophils (×10^9^/L)	188	0.95	0.62, 1.24	0.8
IgE (IU/mL)	188	1.00	0.99, 1.01	0.6
Serum Iron (µg/dL)	188	1.01	0.98, 1.02	0.6
ESR (mm/h)	188	1.02	0.97, 1.06	0.4
CPR (mg/dL)	188	1.02	0.92, 1.07	0.6

^1^ OR = odds ratio, CI = confidence interval. IgA: immunoglobulin A, IgE: immunoglobulin E, Neu: neutrophils, CPR: C-reactive protein, ESR: erythrocyte sedimentation rate.

## Data Availability

Data supporting reported results can be found at corresponding author.
